# Analysis and Enhancements of a Prolific Macroscopic Model of Epilepsy

**DOI:** 10.1155/2016/3628247

**Published:** 2016-04-07

**Authors:** Christopher Fietkiewicz, Kenneth A. Loparo

**Affiliations:** Electrical Engineering and Computer Science Department, Case Western Reserve University, Cleveland, OH 44106, USA

## Abstract

Macroscopic models of epilepsy can deliver surprisingly realistic EEG simulations. In the present study, a prolific series of models is evaluated with regard to theoretical and computational concerns, and enhancements are developed. Specifically, we analyze three aspects of the models: (1) Using dynamical systems analysis, we demonstrate and explain the presence of direct current potentials in the simulated EEG that were previously undocumented. (2) We explain how the system was not ideally formulated for numerical integration of stochastic differential equations. A reformulated system is developed to support proper methodology. (3) We explain an unreported contradiction in the published model specification regarding the use of a mathematical reduction method. We then use the method to reduce the number of equations and further improve the computational efficiency. The intent of our critique is to enhance the evolution of macroscopic modeling of epilepsy and assist others who wish to explore this exciting class of models further.

## 1. Introduction

Significant attention has been given to computational models of epilepsy that simulate the electroencephalogram (EEG) at the level of a neuronal population [1–4]. Such models are referred to by various names such as macroscopic, neural mass, and mean field. These models are capable of synthesizing realistic EEG time series with far less computational effort than that of microscopic models that operate at the scale of single neurons. In addition to being efficient, low-dimensional macroscopic models are also amenable to mathematical analysis methods that can be used to understand key properties of the system being simulated.

Most macroscopic models used in computational neuroscience today are derived, to some extent, from one of three seminal formulations: Freeman [5], Wilson and Cowan [6], and Lopes da Silva et al. [7]. In epilepsy modeling, the approach of Lopes da Silva et al. is particularly prominent and has led to important hypotheses about epileptogenesis and the characteristics of the epileptiform EEG [4].

Wendling et al. have been the most prolific in using the basic approach of Lopes da Silva, with at least 17 different studies during the years 2000–2013. A key feature of their approach is the incorporation of synaptic interactions between specific groups of neurons. This permits the study of a broad class of mechanisms for epileptogenesis that depend on the levels of network excitation and inhibition. Most of their models are direct extensions of the previous work of Jansen et al. [8, 9] that modeled evoked response potentials in human cortical columns.

The earliest model of Wendling et al. used the same structure as Jansen et al. and many of the same parameter values [10]. Wendling et al. qualitatively compared the model to depth-EEG recordings from the human neocortex, hippocampus, and amygdala of patients with temporal lobe epilepsy (TLE) [10–17]. Other models adhered to the same methodology but increased the overall complexity in order to achieve additional dynamical behaviors [18–26].

In the present work, the modeling approach of Wendling et al. is critiqued with regard to theoretical and computational concerns, and enhancements are developed. Specifically, we analyze three aspects of the models: (1) Using dynamical systems analysis, we demonstrate and explain the presence of direct current potentials in the simulated EEG that were previously undocumented. (2) We explain how the system was not ideally formulated for numerical integration of stochastic differential equations. A reformulated system is developed to support proper methodology. (3) We explain an unreported contradiction in the published model specification regarding the use of a mathematical reduction method. We then use the method to reduce the number of equations and further improve the computational efficiency.

## 2. Methods

### 2.1. Mathematical Model

**Figure 1:**
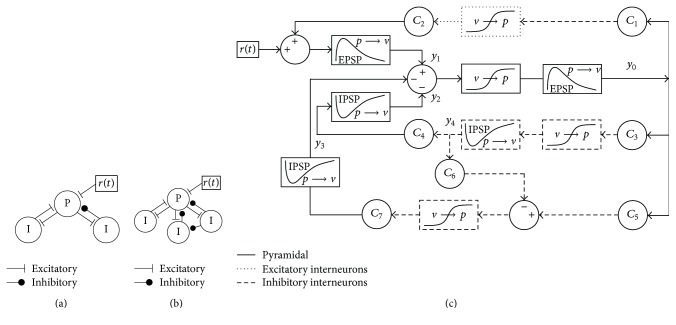
Core models. (a) Initial model [10] showing pyramidal (P) and interneuron (I) subgroups with either excitatory or inhibitory projections. *r*(*t*) is a random input. (b) Extended model [18]. (c) Detailed diagram of computational components for pyramidal cells (solid lines), excitatory interneurons (dotted lines), and inhibitory interneurons (dashed lines). Pulse-to-voltage blocks are labeled “*p* → *v*”, and voltage-to-pulse blocks are labeled “*v* → *p*”. The IPSP block is distinguished from the EPSP blocks by a waveform showing negative deflection.

A basic diagram of the earliest model [10] is provided in Figure 1(a) and shows three neuronal subgroups: excitatory pyramidal cells, excitatory interneurons, and inhibitory interneurons. The present study used an extended version containing four subgroups [18], as shown in Figure 1(b), that contains an additional subgroup of inhibitory interneurons.

Figure 1(c) shows a detailed diagram of the specific computational components of the extended model. A subgroup is a collection of similar but unconnected neurons acting in parallel. Inputs and outputs are represented as firing rates or pulse densities. For each subgroup, two types of conversion blocks work together to transform the input signal into an output signal. The first is a pulse-to-voltage block that represents the process that occurs at a neuronal synapse. An afferent pulse density is converted to postsynaptic potentials (PSPs) that are either excitatory (EPSPs) or inhibitory (IPSPs). Mathematically, this block is a linear transfer function implemented using a differential equation. The second conversion block is a voltage-to-pulse block that translates the summed input voltages into a single representative pulse density. This block is a nonlinear algebraic sigmoidal function.

Subgroups are connected using multiplier constants, labeled *C*_1_–*C*_7_ in Figure 1(c). These constants represent the relative numbers of synaptic connections. Parameters specific to each subgroup include average dendritic time constants and average synaptic gains. The synaptic gains correspond to the relative magnitudes of PSPs and have typically been the only parameters that were studied.

The full equations are shown below, using the original variable indexing [18], and Table 1 lists the model parameters:

(1) y˙0t=y5,

(2) y˙1t=y6,

(3) y˙2t=y7,

(4) y˙3t=y8,

(5) y˙4t=y9,

(6) y˙5t=AaSy1t−y2t−y3t−2ay5t−a2y0t,

(7) y˙6t=Aapt+C2SC1y0t−2ay6t−a2y1t,

(8) y˙7t=BbC4SC3y0t−2by7t−b2y2t,

(9) y˙8t=GgC7SC5y0t−C6y4t−2gy8t−g2y3t,

(10) y˙9t=BbSC3y0t−2by9t−b2y4t,

where *p*(*t*) is a normally distributed random variable with a mean of 90 and a variance of 30,  *a* = 100 s,  *b* = 50 s,  *g* = 350 s,  *C*_1_ = 135,  *C*_2_ = 0.8 × *C*_1_,  *C*_3_ = *C*_4_ = 0.25 × *C*_1_,  *C*_5_ = 0.3 × *C*_1_,  *C*_6_ = 0.1 × *C*_1_,  *C*_7_ = 0.8 × *C*_1_,  *S*(*v*) = 2*e*_0_/[1 + *e* ^*r*(*v*_0_ − *v*)^],  *v*_0_ = 6 mV,  *e*_0_ = 2.5 s^−1^,  *r* = 0.56 mV^−1^. *A*, *B*, and *G* represent average synaptic gains, the values of which are chosen to yield one of several possible types of model output. The model output is defined as *y*_out_ = *y*_1_ − *y*_2_ − *y*_3_.

Dynamical systems analysis was performed, based on the above equations, using an approach described previously [27] for analysis of the initial model of Wendling et al. [10]. To the best of our knowledge, the present study is the first to perform the analysis on the extended model [18]. Equilibrium points were solved numerically using Mathematica (Wolfram Research, Champaign, Illinois) and the MATLAB Optimization Toolbox (The MathWorks, Natick, MA). Stability of selected points was determined using the system Jacobian matrix and computing the corresponding eigenvalues, all using the MATLAB Optimization Toolbox.

### 2.2. Numerical Integration

All simulations were performed using MATLAB (The MathWorks, Natick, MA). Numerical integration was done using a fixed-step forward Euler method. Source code for simulations will be made available publicly on ModelDB (https://senselab.med.yale.edu/modeldb/).

As noted in Results, certain simulations used a stochastic forward Euler numerical integration method [28]. The only equation that contains a random variable is (7). For the purpose of stochastic numerical integration, we redefined *p*(*t*) and (7) as follows:

(11) pt=rth0.00130+90,y6tn+1=y6tn+Aartnh0.00130+hAa90+C2SC1y0tn−2ay6tn−a2y1tn,

where *h* is the Euler integration step size and *r*(*t*) is a normally distributed random variable with a mean of 0 and a variance of 1. The variable *r*(*t*) is introduced so that *h* can be seen as an explicit model parameter for a given simulation. We have chosen a “reference” step size of 0.001, as used in previous studies, such that both the stochastic and classical implementations will be identical for *h* = 0.001 sec.

## 3. Results

We analyze three aspects of the models: (1) Using dynamical systems analysis, we demonstrate and explain the presence of direct current potentials in the simulated EEG that were previously undocumented. (2) We explain how the system was not ideally formulated for numerical integration of stochastic differential equations. A reformulated system is developed to support proper methodology. (3) We explain an unreported contradiction in the published model specification regarding the use of a mathematical reduction method. We then use the method to reduce the number of equations and further improve the computational efficiency.

### 3.1. DC Offset

**Figure 2:**
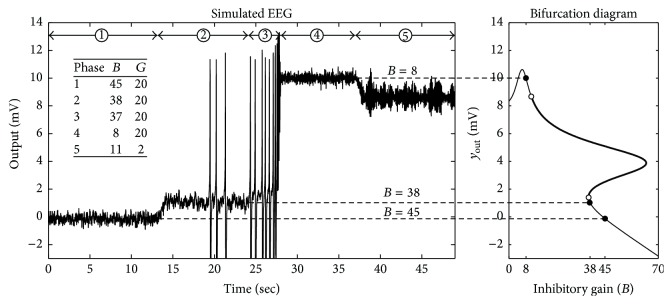
Example of DC offset in the model. (Left) Recreation of simulation of five different dynamical behaviors shown in Wendling et al. [18]. The inset shows values for parameters *B* and *G* that vary for each phase while *A* is fixed at 5. (Right) A bifurcation diagram showing the nullcline that predicts the DC offset based on the inhibitory gain, *B*, for three different values (8, 38, and 45). Filled circles are stable equilibrium points that correspond to similar mean values in the simulated EEG on the left (dashed lines). Open circles indicate bifurcations between stability and instability on the nullcline. The dark line between the open circles represents a continuous region of unstable equilibrium points.

Models are not expected to be perfect replications, but knowledge of the underlying inaccuracies is critical to proper usage [29]. The model analyzed here exhibits a nonzero mean potential that is different for each parameter configuration. To the best of our knowledge, this has never been reported in any studies by Wendling et al. This direct current (DC) offset is demonstrated in Figure 2 which shows a simulation similar to that of Wendling et al. [18]. Using different parameter combinations, the simulation consisted of a progression of five phases of behavior: (1) background activity, (2) sporadic spiking, (3) sustained spiking, (4) gamma activity, and (5) ictal activity. For each phase, the simulation used a unique combination of values for the parameters *B* and *G*, while *A* remained fixed with a value of 5. The inset of Figure 2 shows the values used for *B* and *G*.

The DC offset was present in a predecessor model [9] but did not appear in studies by Wendling et al. that were based on that model. Through personal communication with the author, we learned that this DC potential was removed from simulations by either applying a high-pass filter or subtracting the mean value.

We used dynamical systems analysis to study the observed DC offset values. To the best of our knowledge, the present study is the first to perform such analysis on the model. For an accessible treatment of dynamical systems theory, see Strogatz [30]. For a specific discussion of the analysis of neural models, see Milton et al. [31]. We computed the equilibrium points for Phases 1–4, where only the inhibitory gain *B* was changed. Phase 5 was not studied because it involved an additional parameter change for the inhibitory gain *G*. However, Phases 3 and 5 could be studied in a similar manner.

Using the approach of Grimbert and Faugeras [27], we derived the nullcline for the system $\dot{Y}=f\left(Y\right)=0$. The equilibrium points were calculated by assigning $\dot{Y}=0$ and rearranging the resulting algebraic equations as follows:

(12) y0=AaSy1−y2−y3,y1=Aap+C2SC1y0=Aap+C2SC1AaSy1−y2−y3,y2=BbC4SC3y0=BbC4SC3AaSy1−y2−y3,y3=GgC7SC5y0−C6y4=Gg·C7SC5AaSy1−y2−y3−C6BbSC3AaSy1−y2−y3,y4=BbSC3y0=BbSC3AaSy1−y2−y3.

Note that low-amplitude output fluctuations in the simulation are due to the random drive *p*(*t*). For the dynamical systems analysis, the mean of *p*(*t*) was used (*p* = 90). The model output is defined as *y*_out_ = *y*_1_ − *y*_2_ − *y*_3_. Substituting this into the equations above yields the following combined equation:

(13) yout=Aap+C2SC1AaSyout−BbC4SC3AaSyout−GgC7SC5AaSyout−C6BbSC3AaSyout.

Figure 2 (right) shows the nullcline for the system output *y*_out_ as determined by the above equation. The nullcline includes stable equilibrium points (filled circles) that correspond to similar mean values in the simulated EEG (see dashed lines). A continuous region of unstable equilibrium points (dark line) is also shown in Figure 2 between two bifurcations (open circles).

Table 2 lists the equilibrium points and their stabilities for specific phases. Stability is based on the eigenvalues of the system Jacobian matrix. For stable points, the real parts of all eigenvalues are negative. For unstable points, at least one eigenvalue has a positive real part. Tables 3, 4, and 5 list the eigenvalues that correspond to the eight equilibrium points listed in Table 2. The eigenvalues were computed from the system Jacobian matrix using the MATLAB Symbolic and Optimization Toolboxes (The MathWorks, Natick, MA). For all tables, *y*_out_ corresponds to the ordinate (vertical axis) in Figure 2 (right) and can be used to identify each unique equilibrium point. For stable points, the real parts of all eigenvalues are negative. For unstable points, at least one eigenvalue has a positive real part.

In Figure 2 and Table 2, it can be seen that Phases 1 and 2 each have one stable and two unstable equilibrium points. As *B* decreases, a bifurcation occurs at *B* = 37.3, where a stable point and one unstable point both disappear. This saddle-node bifurcation corresponds to the emergence of a stable limit cycle that is present in Phase 3, where one unstable equilibrium point remains. As *B* decreases further, another bifurcation occurs at which the limit cycle disappears, and one stable equilibrium point remains. We determined that this bifurcation occurs in the range 9.21 < *B* < 9.22, based on a systematic stability analysis of equilibrium points in that region.

Of particular interest in the above analysis is the DC offset that is different for each phase. High-pass filtering is commonly used in EEG acquisition to eliminate DC and improve dynamic range. DC offset in the physiological EEG is still poorly understood, but studies have shown that it coincides with ictal activity in some circumstances. Contrary to the simulation in Figure 2, most studies show that the shift is invariably negative with respect to the baseline [32–35] though the opposite has also been observed [36]. Of these studies, the maximum shift reported was 2.3 mV, in contrast to a shift of 10 mV in the simulations.

Clearly, the model was not designed to accurately simulate the DC shift because electrochemical effects are not directly accounted for. Electrochemical changes are the most likely mechanism responsible for the DC offset that is seen in the EEG. To the best of our knowledge, only one epilepsy model has specifically addressed EEG offset [34, 35]. However, that model is not based on physiology and does not specifically account for electrochemical dynamics. In fact, the authors state that the model output was circumstantially chosen as the sum of two state variables because of its visual resemblance to the EEG [35]. Furthermore, the study does not suggest any physiological significance of the DC offset.

Note that Labyt et al. [22] described studying what they called “stability” properties for a significantly more complex model with twelve neuronal subgroups. However, that analysis was a Monte Carlo procedure in which the term “stable” was used to distinguish nonictal behavior from ictal behavior. It did not address the DC offset, and it was not an analysis of dynamical stability.

### 3.2. Numerical Integration

The literature indicates that the model has historically been simulated as a set of ordinary differential equations. This was verified by source code that is publicly available on ModelDB (https://senselab.med.yale.edu/modeldb/). A problem arises because the system actually consists of stochastic differential equations (SDEs) due to the inclusion of the random variable *p*(*t*) in (7). Classical numerical integration methods are not appropriate for SDEs because they scale random variables by the integration step size. For a practical introduction to SDEs, see [28, 37, 38].

**Figure 3:**
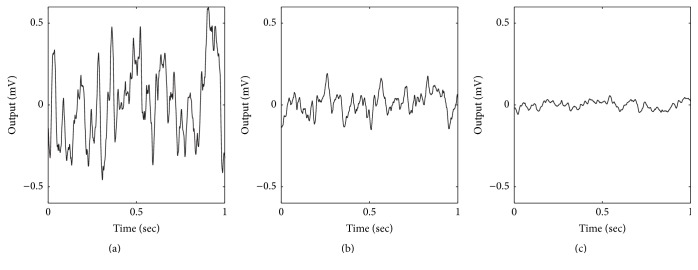
Examples of using the 4th-order Runge-Kutta method, as in Wendling et al. [18]. (a) For integration step size of 1 ms, the variance (*σ* ^2^) of the output is 0.0751. (b) For integration step size of 0.1 ms, *σ* ^2^ is 0.0065. (c) For integration step size of 0.01 ms, *σ* ^2^ is 0.0006. Variances were computed using 10 seconds of output. Model parameters: *A* = 5, *B* = 40, and *G* = 20.

An undesirable consequence is that, as the integration step size becomes smaller (e.g., 1 ms, 0.1 ms, and 0.01 ms), the accuracy of the simulated output actually decreases.  Figure 3 shows examples of simulating the model with a 4th-order Runge-Kutta method, as described in Wendling et al. [18], using different integration step sizes. Notice that the relative peak-to-peak amplitude and variance of the signal continue to decrease as the integration step size decreases, instead of converging to stable values. The variance actually changes by the same order of magnitude as the step size, suggesting a direct relationship between the two. The graphs show 1-second simulations in order to visually compare the similarity in waveform shapes. However, the variances were calculated using 10-second simulations in order to obtain values that were consistent across multiple simulations for the same step size.

**Figure 4:**
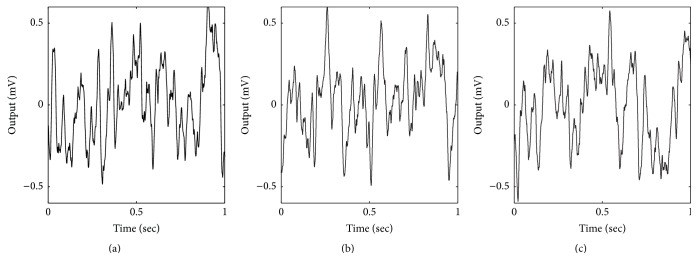
Examples of using forward Euler for SDEs. (a) For integration step size of 1 ms, the variance (*σ* ^2^) of the output is 0.0779. (b) For integration step size of 0.1 ms, *σ* ^2^ is 0.0655. (c) For integration step size of 0.01 ms, *σ* ^2^ is 0.0621. Variances were computed using 10 seconds of output. Model parameters: *A* = 5, *B* = 40, and *G* = 20.

We addressed the issue by reformulating (7) such that the stochastic term is not scaled by the integration step size (see Section 2).  Figure 4 shows examples of using this approach. Using this revised formulation, there is still a slight decrease in variance, but it is greatly improved in comparison to Figure 3. Note that each simulation in Figure 4 is a unique solution because each change in step size involves a different number of random input samples. For a step size of 1 ms, the simulations in Figures 3 and 4 are identical. This is due to a careful reformulation of the equations prior to implementing the SDE numerical method. In the following section, we will provide the reformulation in the context of the full set of equations. Technically, all of the simulations published by Wendling et al. could be duplicated using this approach.

### 3.3. Equation Reduction

**Figure 5:**
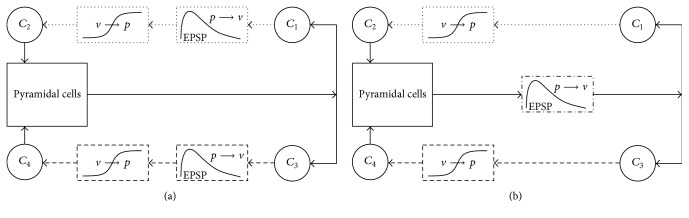
Mathematically equivalent models. (a) Jansen et al. [8] are physiologically accurate with one PSP block for each input into a neuronal subgroup. The same approach was used in Wendling et al. but contradicts the equations. (b) The structure was changed in Jansen and Rit [9] such that two neuronal subgroups share a common PSP block. Cell groups: pyramidal cells (solid lines), excitatory interneurons (dotted lines), and inhibitory interneurons (dashed lines). The EPSP block is shown with dash-dotted lines to indicate that it corresponds to both the excitatory and inhibitory EPSP blocks in (a).

Lastly, we present a discrepancy in the published models that, to the best of our knowledge, has never been addressed. In Wendling et al. [18], the structure of the model can be difficult to interpret because the mathematical definition strayed from the actual physiology being modeled. As described in Methods, each PSP block translates into one differential equation. A careful reading of the 2002 article reveals seven PSP blocks but only five differential equations. The equations actually agreed with an earlier version of the block diagram in Wendling et al. [10], but that diagram was not consistent with the physiological interpretation of the PSP block. Ironically, that diagram was based on earlier studies that also had the same inconsistency [8, 9]. The change that occurred in these earlier studies was that the two conversion blocks within each subgroup were artificially separated. This simplified the model such that multiple subgroups could share the same block. Specifically, the excitatory interneuron group and the inhibitory interneuron group were revised so as to share the same PSP conversion block.  Figure 5 compares a physiologically accurate block diagram with the modified version.

**Figure 6:**
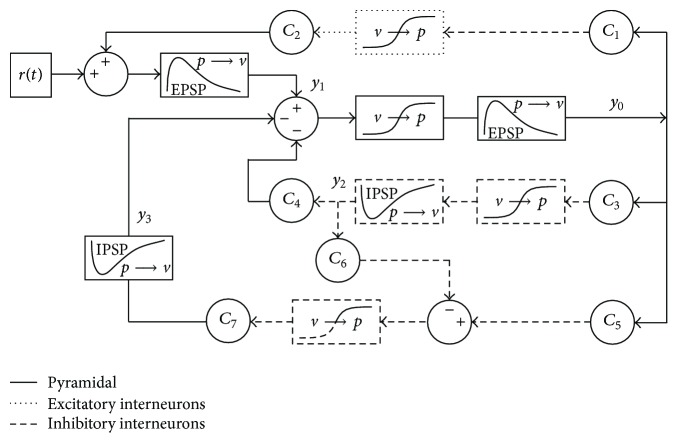
Revised model with additional mathematical reduction. The former *y*_4_ has been renamed as *y*_2_, and the IPSP block for the former *y*_2_ has been removed.

The reduction is reasonable considering that both subgroups share the same input and that the PSP block is modeled as a linear transfer function. The end result is a reduction in the required number of equations. For unknown reasons, a similar reduction was not applied to the dual output paths from inhibitory interneurons whose output was defined as *y*_4_. Such a reduction would increase the efficiency of the formulation further.  Figure 6 shows this reduction in which the former *y*_4_ has been renamed as *y*_2_, and the IPSP block for the former *y*_2_ has been removed.

A reduced set of equations is provided below that contains three major revisions. First, the input to the pyramidal cells now uses the multiplier *C*_4_ (see the equation for the new ${\dot{y}}_{4}$). Second, the variables formerly named *y*_4_ and *y*_9_ have been renamed as *y*_2_ and *y*_6_, respectively. Third, we have separated the stochastic and deterministic terms to enable the proper use of numerical integration methods as described earlier (see the equation for the new ${\dot{y}}_{5}$). The complete system is defined as follows:

(14) y˙0t=y4,

(15) y˙1t=y5,

(16) y˙2t=y6,

(17) y˙3t=y7,

(18) y˙4t=AaSy1t−C4y2t−y3t−2ay4t−a2y0t,

(19) y˙5t=0.001Aaσ2rt+Aam+C2SC1y0t−2ay5t−a2y1t,

(20) y˙6t=BbSC3y0t−2by6t−b2y2t,

(21) y˙7t=GgC7SC5y0t−C6y4t−2gy7t−g2y3t,

where *r*(*t*) is a normally distributed random variable with a mean of 0 and a variance of 1, *σ* ^2^ is the desired input variance, and *m* is the desired input mean. We repeated the simulation shown in Figure 2 and confirmed that the results are identical.

## 4. Discussion

We critiqued a prolific computational modeling approach that has been used for the study of epilepsy. We evaluated three aspects of the models with regard to theoretical and computational concerns, and we developed enhancements to the model formulation. None of the issues that were raised invalidate the published results. However, we feel they are important considerations for other researchers to utilize the models effectively.

First, we demonstrated that a previously unreported DC offset is present in the model and that the offset varies for different parameter configurations. As explained previously, the presence of a DC offset is a well-known characteristic of the physiological EEG that is typically ignored. However, the model was not designed to incorporate any supposed mechanism for this phenomenon. Though the model produces an output that is interpreted as a voltage, the reactive-diffusive process of extracellular ion flow is in no way described by the system. We used dynamical systems analysis to show how the DC offset in the model can be predicted from the equations. Though another model has specifically addressed DC offset [35], no physiological significance was suggested. Future work can explore whether the model correctly describes the phenomenon that is observed in physiological systems.

Second, we described how numerical integration methods may significantly affect the results. Using the published method, the voltage amplitude of the simulated EEG was greatly affected by the integration step size. Methods appropriate for SDEs require a separation of stochastic and deterministic terms. From a practical perspective, this affects whether results are reproducible by other researchers. We provided a reformulation of the equations in order to separate the stochastic and deterministic terms, and we described how this formulation would be implemented using a forward Euler integration method.

Note that there are additional numerical methods available for SDEs. For example, a stochastic Runge-Kutta method exists [28], but it is only applicable when the random variable is multiplicative with respect to a state variable. In the present system, the term with the random variable does not contain a state variable. Two significantly different integration approaches can be found in [39, 40]. The latter study is actually based on the EEG model in [9]. However, these approaches cannot be compared directly to classical methods in the same manner as we have done here. Future work can evaluate the efficiency of these alternative integration methods for the present model.

Third, we discussed a mathematical reduction that led to a contradiction between system diagrams and the equations in the literature. We further proposed a modification to improve the efficiency of the equations by applying the same mathematical reduction to a different part of the model. Though the reduction is mathematically equivalent to the longer form, it is an important conceptual modification because it contradicts actual physiological structure.

The intent of our critique is to enhance the evolution of macroscopic modeling of epilepsy and assist others who wish to explore this exciting class of models further. Just as modeling is only one research tool among many, macroscopic modeling is merely one of many levels of modeling that are needed to study a system like the brain that exhibits complexities at many temporal and spatial scales. Microscopic models of large networks may require significant computing power, but macroscopic models can usually be simulated using common office computing equipment. As we have demonstrated here, low-dimensional models also allow for rigorous mathematical analysis in order to better understand the mechanisms behind dynamical behavior. These advantages can benefit epilepsy research as well as neuroscience in general.

## Figures and Tables

**Table 1:** Model parameters from Wendling et al. [18].

Param.	Interpretation	Value
*A*	Average excitatory synaptic gain	5 mV
*B*	Average slow inhibitory synaptic gain	See Figure 2
*G*	Average fast inhibitory synaptic gain	See Figure 2
*a*	Dendritic average time constant in the feedback excitatory loop	100 s^−1^
*b*	Dendritic average time constant in the slow feedback inhibitory loop	50 s^−1^
*g*	Somatic average time constant in the fast feedback inhibitory loop	350 s^−1^
*C*_1_, *C*_2_	Mean number of synaptic contacts in the excitatory feedback loop	135, 0.8 × *C*_1_
*C*_3_, *C*_4_	Mean number of synaptic contacts in the slow feedback inhibitory loop	0.25 × *C*_1_
*C*_5_, *C*_6_	Mean number of synaptic contacts in the fast feedback inhibitory loop	0.3 × *C*_1_, 0.1 × *C*_1_
*C*_7_	Mean number of synaptic contacts between slow and fast inhibitory interneurons	0.8 × *C*_1_

**Table 2:** Equilibrium points and their stabilities for specific phases. *y*_out_ = *y*_1_ − *y*_2_ − *y*_3_. For each equilibrium point, *y*_5_ = *y*_6_ = *y*_7_ = *y*_8_ = *y*_9_ = 0. Stability is based on eigenvalues in Tables 3, 4, and 5.

Phase	*B*	Stability	*y*_out_	*y*_0_	*y*_1_	*y*_2_	*y*_3_	*y*_4_
1	45	Stable	−0.124	0.008	6.097	5.882	0.339	0.174
		Unstable	2.526	0.031	11.777	8.962	0.290	0.266
		Unstable	5.087	0.094	30.864	25.749	0.028	0.763

2	38	Stable	1.018	0.014	7.037	5.600	0.419	0.166
		Unstable	1.781	0.022	8.553	6.358	0.415	0.188
		Unstable	5.416	0.105	31.220	25.768	0.036	0.764

3	37	Unstable	5.466	0.106	31.254	25.750	0.037	0.763

4	8	Stable	10.004	0.226	31.500	19.258	2.238	0.571

**Table 3:** Eigenvalues for Phase 1, *B* = 45.

*y*_out_	−0.124	2.53	5.09

Stability	Stable	Unstable	Unstable

*λ*_0_	−178.1	−241.4	−181.9
*λ*_1_	−65.9	−60.7	−78.5
*λ*_2_	−50.0	−50.0	−50.0
*λ*_3_	−50.0	−50.0	−50.0
*λ*_4_	−24.0 + 24.5*i*	−30.3	−134.4 + 98.9*i*
*λ*_5_	−352.4 + 24.5*i*	48.7	15.9 − 78.8*i*
*λ*_6_	−24.0 − 24.5*i*	−99.5 − 156.0*i*	−134.4 − 98.9*i*
*λ*_7_	−352.4 − 24.5*i*	−358.7 − 42.7*i*	−351.3 − 19.0*i*
*λ*_8_	−101.7 − 83.1*i*	−99.5 + 156.0*i*	15.9 + 78.8*i*
*λ*_9_	−101.7 + 83.1*i*	−358.7 + 42.7*i*	−351.3 + 19.0*i*

**Table 4:** Eigenvalues for Phase 2, *B* = 38.

*y*_out_	1.02	1.78	5.42

Stability	Stable	Unstable	Unstable

*λ*_0_	−197.9	−216.7	−142.3
*λ*_1_	−63.2	−61.8	−84.0
*λ*_2_	−50.0	−50.0	−50.0
*λ*_3_	−50.0	−50.0	−50.0
*λ*_4_	−100.0 + 107.1*i*	−25.0	−155.6 + 91.8*i*
*λ*_5_	−14.2 − 14.0*i*	17.1	−351.5 − 21.4*i*
*λ*_6_	−100.0 − 107.1*i*	−357.6 − 42.5*i*	−155.6 − 91.8*i*
*λ*_7_	−355.2 − 35.9*i*	−99.2 − 129.5*i*	20.3 + 89.2*i*
*λ*_8_	−355.2 + 35.9*i*	−99.2 + 129.5*i*	20.3 − 89.2*i*
*λ*_9_	−14.2 + 14.0*i*	−357.6 + 42.5*i*	−351.5 + 21.4*i*

**Table 5:** Eigenvalues for Phase 3 (*B* = 37) and Phase 4 (*B* = 8).

	Phase 3 *B* = 37	Phase 4 *B* = 8
*y*_out_	5.47	10.00

Stability	Stable	Unstable

*λ*_0_	−137.8	−172.0
*λ*_1_	−84.7	−59.3
*λ*_2_	−50.0	−50.0
*λ*_3_	−50.0	−50.0
*λ*_4_	−157.9 + 91.9*i*	−352.2 + 23.6*i*
*λ*_5_	−351.6 − 21.9*i*	−32.6 + 9.8*i*
*λ*_6_	−157.9 − 91.9*i*	−352.2 − 23.6*i*
*λ*_7_	20.7 + 90.2*i*	−99.5 − 77.1*i*
*λ*_8_	20.7 − 90.2*i*	−32.6 − 9.8*i*
*λ*_9_	−351.6 + 21.9*i*	−99.5 + 77.1*i*

## Additional Points

Source code for simulations will be made available publicly on ModelDB (https://senselab.med.yale.edu/modeldb/).
